# A196 MATERNAL AND NEONATAL OUTCOMES IN PREGNANT WOMEN WITH IBD TREATED WITH USTEKINUMAB OR RISANKIZUMAB: A CASE SERIES

**DOI:** 10.1093/jcag/gwae059.196

**Published:** 2025-02-10

**Authors:** K Li, H Nabavian, S Eisen, K O’Connor, V Srikanth, V Huang

**Affiliations:** University of Toronto Temerty Faculty of Medicine, Toronto, ON, Canada; University of Toronto Temerty Faculty of Medicine, Toronto, ON, Canada; University of Toronto Temerty Faculty of Medicine, Toronto, ON, Canada; Sinai Health, Toronto, ON, Canada; Sinai Health, Toronto, ON, Canada; Sinai Health, Toronto, ON, Canada

## Abstract

**Background:**

Biologics are an important therapy for patients with moderate to severe IBD. These include Ustekinumab (UST) – anti-IL-12/23, and the newer biologic Risankizumab (RIS) – anti-IL23. For UST, the PIANO registry and some cohort studies found no significant difference in maternal/neonatal outcomes compared to controls. However, the EPI-MERES registry found an increase in likelihood of small for gestational age births, and one study reported a slower initial postnatal growth rate for in-utero exposed infants. RIS currently has limited evidence in pregnancy.

**Aims:**

We present a case series of maternal and neonatal outcomes of UST and RIS treated pregnant patients with IBD.

**Methods:**

We conducted a single-centre case series including patients treated with UST and RIS at any point during pregnancy enrolled between Jan 1, 2017 and Jul 31, 2024. Data was collected on maternal outcomes including live birth, miscarriage, delivery method, and pregnancy-related complications. Neonatal outcomes included preterm birth, small for gestational age (SGA), large for gestational age (LGA), congenital anomalies, NICU admission, APGAR scores, and neonatal infection.

**Results:**

There were 23 UST patients – 21 (91.3%) had live births and 2 (8.7%) had miscarriages. Of the 21 that delivered, there were 12 (57.1%) vaginal deliveries, 8 (38.1%) elective c-sections, and 1 (4.8%) emergency c-section due to placental insufficiency. One patient had gestational diabetes, 1 had pre-eclampsia, but there were no preterm premature rupture of membranes or intrauterine growth restrictions (IUGR). Of the 21 neonates, 3 (14.3%) were preterm, 1 (4.8%) was SGA, 3 (14.3%) were LGA, and 1 unknown. There were no congenital anomalies. Five (23.8%) infants were admitted to the NICU after birth and 1 (4.8%) was reported to have a postnatal infection within 3 months. Of the 12 infants with known APGAR scores, 10 had scores ≥7 at 1 minute, and 2 were <7. At 5 minutes, all scores were ≥7.

Of the 2 RIS patients, 1 was exposed to RIS throughout the entire pregnancy and the other was given a dose during the 1st and 3rd trimester only. Both pregnancies resulted in live births with 1 vaginal delivery and 1 elective c-section. One neonate was LGA. One had an APGAR score of 8 at 1 minute and 9 at 5 minutes. The other infant scored 9 at 1 minute and the score at 5 minutes was not documented. There were no preterm births, congenital anomalies, or pregnancy-related complications.

**Conclusions:**

In our case series of UST and RIS exposed pregnancies, there were no significant adverse maternal or neonatal outcomes. Further data is necessary to better characterize the safety of both medications.

**Funding Agencies:**

None

